# Malnutrition Defined by the Global Leadership Initiative on Malnutrition (GLIM) Criteria in Hospitalized Patients with Ulcerative Colitis and Its Association with Clinical Outcomes

**DOI:** 10.3390/nu15163572

**Published:** 2023-08-14

**Authors:** Wei Wei, Pengguang Yan, Fang Wang, Xiaoyin Bai, Jing Wang, Jingnan Li, Kang Yu

**Affiliations:** 1 Department of Clinical Nutrition, Peking Union Medical College Hospital, Chinese Academy of Medical Science and Peking Union Medical College, Beijing 100730, China; weiwei@pumch.cn (W.W.); wangfang98@pumch.cn (F.W.); 2 Department of Gastroenterology, Peking Union Medical College Hospital, Chinese Academy of Medical Science and Peking Union Medical College, Beijing 100730, China; yanpengguang@pumch.cn (P.Y.); baixiaoyin@pumch.cn (X.B.); 13910514866@139.com (J.W.)

**Keywords:** ulcerative colitis, the Global Leadership Initiative on Malnutrition, malnutrition, disease activity

## Abstract

(1) Background: The Global Leadership Initiative on Malnutrition (GLIM) was published in 2019, and its application has been explored in several diseases. However, the data on malnutrition based on the GLIM in ulcerative colitis (UC) patients are sparse. (2) Methods: This single-center, retrospective cohort study included 605 hospitalized UC patients. Demographics and clinical data were collected from electronic medical records. Nutritional Risk Screening 2002 (NRS 2002) was used as a screening tool, and malnutrition was diagnosed according to the GLIM criteria. The skeletal muscle area of the third lumber cross-section in abdominal computed tomography was used to evaluate muscle mass within one week before or after admission. (3) Results: The prevalence of malnutrition was 64.1% in this cohort, and the prevalences were 34.2, 57.7, and 86.7% in UC patients with mild, moderate, and severe disease activity, respectively. Malnourished patients tended to need surgical treatment (*p* = 0.080) and had a 2.4 times greater risk of opportunistic infection. The multivariate logistic regression analysis showed that UC patients with malnutrition had a 1.7-fold increased risk of readmission. (4) Conclusions: Nutritional problems deserve more attention in hospitalized UC patients. Malnutrition identified through the GLIM criteria was associated with opportunistic infection, tended to be associated with surgical treatment, and showed a prognosis value for readmission.

## 1. Introduction

Inflammatory bowel disease (IBD) is an idiopathic immune-mediated inflammatory disorder, mainly represented by ulcerative colitis (UC) and Crohn’s disease (CD). The incidence of IBD has gradually increased in China during the last three decades, reaching 3.01/100,000 person-years, with a prevalence of 47.06/100,000 [1]. Malnutrition can occur in IBD patients due to a combination of factors, including reduced intake, loss of appetite, increased basal metabolic rate caused by inflammation, and malabsorption caused by impaired intestinal barrier integrity, accelerated intestinal peristalsis, decreased intestinal epithelial transport function, and overgrowth of intestinal bacteria [2]. The reported prevalence of IBD patients at high risk of malnutrition is between 28 and 67% [3]. The ESPEN guideline on clinical nutrition in IBD recommends malnutrition screening for all IBD patients at diagnosis and thereafter [4]. However, compared to CD patients, the nutritional status of UC patients has received less attention when analyzing the nutritional status of IBD patients in previous studies [3], probably owing to the viewpoint that the colorectum is the chief target organ of UC, and the nutritional status can be less affected.

Previous studies have defined malnutrition in IBD patients according to different criteria, such as the European Society for Clinical Nutrition and Metabolism (ESPEN) criteria, the Subjective Global Assessment (SGA), the ICD-9 code for malnutrition, and a body mass index (BMI) < 18.5 kg/m^2^ [5]. In 2019, the Global Leadership Initiative on Malnutrition (GLIM) was published to build a global consensus around core diagnostic criteria for malnutrition in adults in clinical settings [6]. Several studies have explored the relationship between disease prognosis and malnutrition based on the GLIM. For example, GLIM-defined malnutrition was independently correlated with mortality in chronic liver disease or cancer patients [7,8]. However, information about the application of the GLIM criteria in UC patients and the association between malnutrition based on GLIM and prognosis is limited.

Therefore, the present study analyzed the prevalence of malnutrition according to the GLIM criteria in a retrospective cohort of hospitalized UC patients and compared the clinical characteristics between patients with and without malnutrition, exploring the clinical application value of GLIM in UC patients.

## 2. Materials and Methods

### 2.1. Study Population

This retrospective single-center study was conducted at Peking Union Medical College Hospital (PUMCH). Hospitalized UC patients were enrolled from November 2014 to August 2022. The inclusion criteria were: (1) 18–70 years old; (2) diagnosed with active UC; and (3) having complete medical records. The exclusion criteria were: (1) comorbidity in other major organs (e.g., heart, lung, liver, and kidney) or endocrine, hematological, and auto-immune diseases other than UC; (2) comorbidity related to neuromuscular or orthopedic diseases, such as myasthenia gravis, Parkinson’s disease, and fracture, which can influence muscle mass; (3) history of cancer or cancer-related comorbidity; (4) admission for diseases other than UC; and (5) pregnant or lactating female. This study was approved by the Ethics Committee of PUMCH (No. I-22PJ700).

### 2.2. Data Collection

The data of enrolled patients were obtained from their medical records, and the collected variables included age, sex, height, weight at admission, non-volitional weight loss, the percentage of food intake reduction and the use of enteral or parenteral nutrition, disease duration, age at diagnosis, length of stay, extra-intestinal manifestations, and surgical treatment. Disease activity was defined according to the modified Truelove and Witts’ criteria. Disease distribution was classified into E1, E2, and E3, based on the Montreal classification. Extra-intestinal manifestations (EIMs) were recorded at the first admission and during follow-up. Laboratory indices, including hemoglobin (HGB), serum albumin (ALB), high-sensitivity C-reactive protein (hsCRP), and prealbumin (PA), were recorded at admission. The opportunistic infections among patients were analyzed: cytomegalovirus (CMV) pp65 antigenemia, positive CMV DNA viral load in blood, a positive finding of serum CMV-specific immunoglobulin M (IgM) antibodies, or CMV detection on colonic mucosal specimens indicated CMV reactivation; positive finding of enzyme immunoassays (EIAs) for *Clostridioides difficile* (*C. difficile*) toxins A and B indicated *C. difficile* infection; and the results of stool tests of other opportunistic pathogens such as fungus, mycobacterium tuberculosis, and non-tuberculosis were also recorded. Each patient was enrolled only once. Meanwhile, for patients hospitalized more than once due to UC recurrence or exacerbation, the above variables were collected according to their first admission; the number of readmissions, the dates of readmissions, and information on extra-intestinal manifestations and surgery treatments after their first discharge were recorded until April 2023.

### 2.3. Assessment of Nutritional Status

According to the GLIM criteria, we first used Nutritional Risk Screening 2002 (NRS2002) to identify patients with nutritional risk. Then, the diagnosis of malnutrition in these patients was made based on at least one of the three phenotypic criteria (weight loss, low BMI, and reduced muscle mass) and at least one of the two etiologic criteria (reduced food intake/assimilation and disease burden/inflammation). The criteria for weight loss were 5–10% within the past six months or 10–20% beyond six months, and the Asian criteria of low BMI were <18.5 kg/m^2^ if <70 years and <20 kg/m^2^ if >70 years [6]. If neither BMI nor weight loss met the criteria, the skeletal muscle area (SMA) at the L3 cross-section of the abdominal CT within one week before or after admission was measured with the Syngo.via software (Siemens Healthineers, Forchheim, Germany), using a muscle-specific Hounsfield unit (HU) range between −29 and +150. The skeletal muscle index (SMI, cm^2^/m^2^), the adjustment of the SMA for height^2^, was used to evaluate muscle mass, with cut-off values of 34.9 cm^2^/m^2^ for women and 40.8 cm^2^/m^2^ for men [9]. Overweight was defined as BMI ≥ 24 kg/m^2^ and <28 kg/m^2^, and obesity was defined as BMI ≥ 28 kg/m^2^ [10].

### 2.4. Statistical Analysis

Demographical and clinical parameters are presented as the mean ± standard deviation (SD) if normally distributed or the median (Q1, Q3) if not. Comparisons between groups were performed using independent sample *t*-test or nonparametric Mann–Whitney U-test. Qualitative data were analyzed using the χ^2^ test, with a two-sided *p* < 0.05 as statistically significant. Multivariate logistic regression analysis was used to identify the risk factors for readmission, and the odds ratio (OR) and 95% confidence interval (CI) were calculated. Statistical analyses were performed using SPSS version 23.0 (SPSS Inc., Chicago, IL, USA) and GraphPad Prism version 6.0 (GraphPad Software Inc., San Diego, CA, USA).

## 3. Results

### 3.1. Demographics and Clinical Characteristics

**Figure 1:**
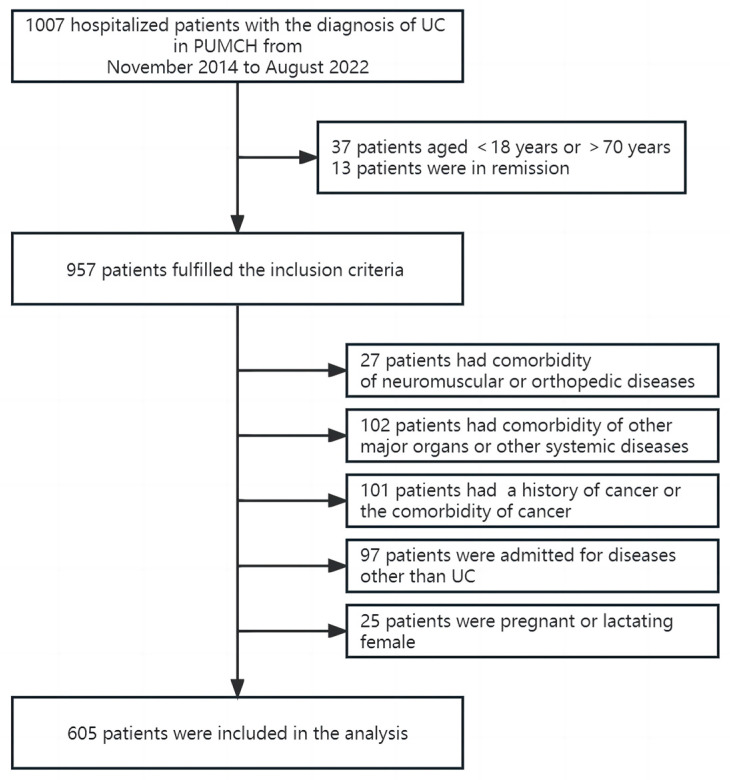
Flowchart for inclusion and exclusion of patients.

There were 1007 hospitalized patients with the diagnosis of UC, and 605 patients with active UC were included in the final analysis (Figure 1). Demographics and clinical data are presented in Table 1. Eighty-five (14.0%) patients were overweight, and eighteen (3.0%) were obese. Seventy-nine (13.1%) patients were treated with surgery at the first hospitalization or during the follow-up period. A total of 101 (16.7%) patients had at least one extra-intestinal manifestation.

### 3.2. Prevalence of Malnutrition in UC Patients

In total, 445 patients (73.6%) were at nutritional risk based on NRS-2002, and 388 patients among them were diagnosed with malnutrition, according to the GLIM criteria. Thus, the prevalence of malnutrition in UC patients was 64.1% in this study. Regarding the phenotypic criteria of GLIM, 158 patients (40.7% of patients with malnutrition and 26.1% of all patients) had low BMI (<18.5 kg/m^2^), and 209 (53.9% of patients with malnutrition and 34.5% of all patients) had non-volitional weight loss, despite a BMI ≥ 18.5 kg/m^2^. Moreover, 21 patients (5.4% of patients with malnutrition and 3.5% of all patients) had reduced muscle mass, while their BMI and weight loss did not reach the GLIM criteria. Hence, among the 447 patients with a BMI ≥ 18.5 kg/m^2^, 230 (51.5%) were malnourished, based on the GLIM.

The age, age at diagnosis, and duration of follow-up did not differ significantly between patients with and without malnutrition. As expected, the disease activity of patients with malnutrition was significantly higher than those without malnutrition (*p* < 0.001) (Table 1). The prevalence of malnutrition increased with disease activity, representing 34.2% (39/114), 57.7% (153/265), and 86.7% (196/226) in patients with mild, moderate, and severe disease activity, respectively. Interestingly, the disease duration of patients with malnutrition was significantly shorter, compared to those without malnutrition (*p* < 0.001) (Table 1). Additionally, the proportion of patients with severe disease activity was significantly higher in patients with a disease duration ≤ 3 years, compared to those with a disease duration > 3 years (43.2% vs. 31.9%, *p* = 0.004).

Malnutrition was differently distributed between younger and older patients. In patients aged 18–50 years, the prevalence of malnutrition was 71.2%, while in patients over 50 years, the prevalence of malnutrition was 49.7% (*p* < 0.001). The proportion of patients with severe disease activity was also significantly higher in patients aged 18–50, compared to those over 50 (40.1% vs. 31.6%, *p* = 0.043).

### 3.3. Nutritional Support

Among the 605 patients, 338 (55.9%) received nutrition support during hospitalization, comprising 178, 130, and 30 patients with severe, moderate, and mild disease activity, respectively. The proportion of patients receiving parenteral nutrition (alone or with enteral nutrition) during hospitalization was 16.4% (99/605). In patients with malnutrition, 68.3% (265/388) received nutritional support. Meanwhile, this rate was 33.6% (73/217) in patients without malnutrition (*p* < 0.001). In malnourished patients with severe, moderate, and mild disease activity, the proportions of nutritional support were 80.6% (158/196), 58.8% (90/153), and 43.6% (17/39), respectively.

### 3.4. Associations between Malnutrition and Prognosis in UC Patients

#### 3.4.1. Extra-Intestinal Manifestations

Sixty-eight (17.5%) and thirty-three (15.2%) patients had extra-intestinal manifestations in the malnutrition and non-malnutrition groups, respectively. However, no significant difference was detected.

#### 3.4.2. Surgical Treatment

The proportion of patients treated with surgery was significantly higher in the malnutrition group compared to the non-malnutrition group [65 (16.8%) vs. 14 (6.5%), *p* < 0.001]. After adjusting for disease activity, patients with malnutrition tended to need surgery more than those without, but no statistical significance was detected (*p* = 0.080).

#### 3.4.3. Opportunistic Infections

Furthermore, we found that 153 (25.3%) UC patients suffered from opportunistic infections, including 73 patients with CMV reactivation, 46 patients with *C. difficile* infection, 29 patients with both CMV reactivation and *C. difficile* infection, 2 patients with intestinal fungal infection (*Candida* species), 1 patient with both CMV reactivation and fungal infection (*Candida* species), and 2 patients with intestinal non-tuberculosis mycobacteria infection. The prevalences of CMV reactivation and *C. difficile* infection in UC patients were 16.7 and 12.4%, respectively. Among patients with opportunistic infections, 126 (32.5%) patients were in the malnutrition group, and 27 (12.4%) were in the non-malnutrition group (*p* < 0.001). After adjusting for disease activity, patients with malnutrition had a 2.4 times greater risk of opportunistic infections than those without (OR = 2.391, 95% CI: 1.470–3.888).

#### 3.4.4. Length of Stay

The length of stay was significantly longer in patients with malnutrition compared to those without malnutrition [18 (12, 26) vs. 9 (6, 15), *p* < 0.001]. Specifically, in patients with mild and moderate disease activity, the length of stay was significantly longer in malnourished patients compared to well-nourished patients [mild: 11 (8, 13) days vs. 7 (6, 12) days, *p* = 0.014; moderate: 15 (10, 21) days vs. 9 (7, 15) days, *p* < 0.001]. The length of stay tended to be longer in malnourished patients with severe UC, yet this difference did not reach statistical significance [22 (15, 29) days vs. 18 (11, 28) days, *p* = 0.100].

#### 3.4.5. Readmission

Multivariate logistic regression was used to investigate the factors that might increase the risk of readmission in UC patients. The patients who underwent surgery during the first hospitalization and those who were hospitalized only once in our hospital and did not follow up in the gastroenterology outpatient department after discharge were excluded. Among the 516 patients enrolled, 205 experienced readmissions, and patients with malnutrition in their first admission experienced a significantly shorter duration between the first and second admission than those without malnutrition [206 (90, 395) days vs. 374 (162, 805) days, *p* < 0.001]. Univariate analysis was performed on the data at the first admission, showing that the disease activity and the prevalence of malnutrition were significantly higher (both *p* < 0.001) in patients with readmission, and the proportion of patients with extra-intestinal manifestations tended to be higher (*p* = 0.066) in patients with readmission. In contrast, the differences in disease extent and the proportion of patients suffering from opportunistic infection were insignificant (Table 2). Then, multivariate logistic regression analysis showed that UC patients with malnutrition had a 1.7-fold increased risk of readmission, regardless of age and gender. Disease activity was also a risk factor for readmission (Table 3).

### 3.5. Associations between Malnutrition and Laboratory Indices in UC Patients

**Figure 2:**
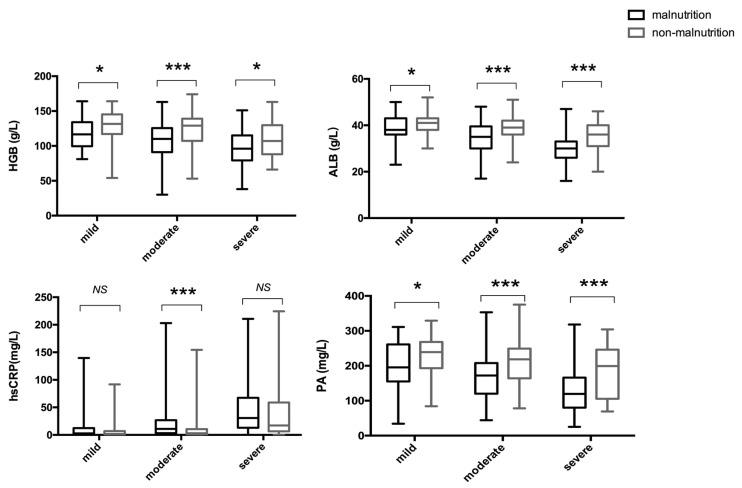
The comparison of laboratory indices between patients with and without malnutrition. UC patients were subdivided based on disease activity and nutritional status. * *p* < 0.05, *** *p* < 0.001, NS, no significance. HGB, hemoglobin; ALB, serum albumin; hsCRP, high-sensitivity C-reactive protein; PA, prealbumin.

All patients underwent a blood routine and a serum albumin test within 24 h after admission; hsCRP and PA tests were conducted on 600 and 563 patients within 24 h after admission, respectively, covering more than 90% of patients. As expected, HGB, ALB, and PA were significantly lower in the malnutrition group, and hsCRP was significantly higher in the malnutrition group (all *p* < 0.001). However, the results were inconsistent among patients with different disease activity levels. In the 114 patients with mild disease activity, HGB, ALB, and PA were significantly lower in patients with malnutrition (*p* = 0.014 for HGB, *p* = 0.044 for ALB, and *p* = 0.033 for PA), whereas the hsCRP did not differ (*p* = 0.256). In the 265 patients with moderate disease activity, HGB, ALB, and PA were significantly lower (all *p* < 0.001), while hsCRP was significantly higher (*p* < 0.001) in patients with malnutrition. Among the 226 patients with severe disease activity, HGB, ALB, and PA were significantly lower (*p* < 0.001 for ALB and PA, and *p* = 0.017 for HGB) in patients with malnutrition; similar to the mild group, hsCRP showed no significant difference (*p* = 0.110) (Figure 2).

## 4. Discussion

To the best of our knowledge, this is the first study to explore the prevalence of malnutrition based on the GLIM criteria in hospitalized UC patients with a relatively large sample size in China. In the present cohort, 73.6% of UC patients had nutritional risk based on NRS-2002, and 64.1% of UC patients had malnutrition. In severe UC patients, the prevalence of malnutrition reached over 80%. Malnourished UC patients had a significantly longer length of stay, a 2.4 times greater risk of having an opportunistic infection, and tended to need surgical treatment, compared with well-nourished ones. Moreover, malnourished patients had a 1.7-fold times greater risk of readmission due to UC recurrence or exacerbation, and the period between the first and second hospitalization was significantly shorter in malnourished patients than in well-nourished patients.

The prevalence of malnutrition in UC has been investigated in several studies based on different diagnostic criteria. A small-sample-sized study (including 48 CD and 25 UC patients) by Zhang et al. showed that the prevalences of malnutrition were 58.90% in hospitalized IBD patients, and the prevalences of malnutrition in CD and UC were 60.42 and 56.00%, respectively, according to the GLIM criteria [11]. A multi-center study with mainly hospitalized patients reported that the prevalence of malnutrition was 49.5% in IBD patients, with 57.0% in CD patients and 38.8% in UC patients, based on the ESPEN criteria [12]. Herein, among the 420 UC patients, in those with quiescent or mildly active UC (34.5% of the UC cohort) and moderately to severely active UC (65.5% of the UC cohort), the prevalences of malnutrition were 22.1 and 47.6%, respectively [12]. Another multi-center study used both the GLIM and ESPEN criteria in UC patients and found that 32.8 and 27.9% of hospitalized UC patients were malnourished, respectively, but the sample size was also relatively small (61 UC patients); additionally, 31.2% of the patients had mild disease activity [13]. The prevalence of malnutrition in UC outpatients is lower than in hospitalized patients, as evidenced by a recently published study with 336 UC outpatients enrolled, reporting a malnutrition prevalence of 24.40%, based on the ESPEN criteria [14]. This study detected a higher prevalence of malnutrition in hospitalized UC patients than in the studies mentioned above. On the one hand, the proportion of patients with mild disease activity (18.8%) was much lower, indicating that this cohort had a higher disease burden and inflammatory conditions. The prevalence of malnutrition increases with disease activity; therefore, the distribution of disease activity in a cohort is an important factor influencing malnutrition prevalence. On the other hand, the ESPEN criteria are more stringent than the GLIM. A patient with a BMI lower than 18.5 kg/m^2^ can be diagnosed with malnutrition by both the ESPEN and GLIM criteria. However, if the BMI is ≥18.5 kg/m^2^, the GLIM requires weight loss of over 5% or reduced muscle mass, which can be proved by any validated body composition measuring techniques, such as fat-free mass index (FFMI) measured by dual-energy absorptiometry (DXA)/bioelectrical impedance analysis (BIA), muscle mass evaluated by CT/MRI, and even calf circumference, while the ESPEN criteria require weight loss over 5%, combined with reduced FFMI or reduced BMI (lower than 20 kg/m^2^ for patients under 70 years or lower than 22 kg/m^2^ for patients over 70 years). Moreover, the acceptable weight loss phase is also longer in the GLIM than in the ESPEN criteria (6 months vs. 3 months). A study on geriatric rehabilitation patients demonstrated that the prevalence of malnutrition was 52.0% based on the GLIM, while it was only 12.6% based on the ESPEN criteria [15]. The high sensitivity of the diagnosis of malnutrition is one of the objectives of GLIM, which is to identify all patients who have nutritional concerns and need nutritional support and to provide them with a reimbursable diagnosis of malnutrition [16].

BMI is always a marker of interest when evaluating nutrition status. The incidence of low BMI in UC inpatients was inconsistent in previous studies, with several studies reporting incidences as low as 10.3% [17] and 13.1% [13] and others reporting incidences as high as 30.2% [12] and 44.0% [11]. Here, the incidence of low BMI in the cohort was 26.1%. Notably, in patients with BMI ≥ 18.5 kg/m^2^, 51.5% had malnutrition, suggesting that a considerable part of undernourished patients will be neglected if we only use the BMI as a nutritional indicator. In malnourished patients, 40.7% had low BMI, while 53.9% experienced drastic weight loss, though their BMI was still over 18.5 kg/m^2^, and these patients accounted for over 1/3 of all UC patients. It was reported that in newly diagnosed UC patients, 32.4% experienced a weight loss of more than 5% of the initial weight in the three months before UC diagnosis [18]. Several factors can contribute to the weight loss of UC patients. First, active UC can increase resting energy expenditure [19]. Second, due to enhanced gastrocolic reflex [20] and the high inflammation level of the gut, food intake is often regarded as a trigger of symptoms by patients. Thus, food restriction is common among UC patients to reduce abdominal pain, cramps, and diarrhea [21], leading to negative energy and nitrogen balances. Additionally, 55.7% of UC patients have decreased appetite during a disease flare [22]. On the other hand, the prevalence of overweightand obesity in western IBD patients was approximately 20–30% during the last decade [23], and a European study in 2019 reported that nearly half of IBD patients attending outpatient clinics were overweight or obese (the prevalence of overweight was 29%, and obesity was 18%) [24]. The prevalence of obesity was 3.0% in the UC population of the present study, consistent with a previous study showing that 3.3% of UC patients were obese, while the prevalence of overweight was 14.0% in our study, lower than the result in that study (27.9%) [13]. Given that the prevalences of overweight and obesity have increased over the past decades in the Chinese population [10], it is reasonable to speculate that IBD patients with low BMI will be reduced, and more attention should be paid to body composition when evaluating the nutrition status of patients in the future. Reduced muscle mass, a phenotypic criterion of GLIM, is an indicator of nutritional status from the perspective of body composition. A systematic review reported that 41.6% of IBD patients had sarcopenia [25], indicating that quite a part of IBD patients have reduced muscle mass and function. However, compared to the accessibility of BMI and non-volitional weight loss, muscle mass measurements by BIA, DXA, CT, or MRI are not always available. In the present study, UC patients who had reduced muscle mass while their BMI and weight loss did not reach the criteria in GLIM only accounted for 5.4% of patients with malnutrition, indicating that the criteria regarding BMI and weight loss can help identify the most of undernourished UC patients. Calf circumference has been used in diagnosing sarcopenia in IBD patients [26,27], and its value in identifying malnutrition in IBD patients deserves further study.

In this study, undernourished patients had a significantly shorter disease duration than well-nourished ones. Meanwhile, the prevalence of malnutrition was significantly higher in younger patients (18–50 years) compared to older ones. Considering that the prevalence of malnutrition increased with the disease activity, the significantly higher proportions of patients with severe disease activity in the population of 18–50-year-olds and in those with a disease duration of less than 3 years can be important contributors to the higher prevalence of malnutrition. A previous study also supported the shorter disease duration and younger age of malnourished IBD patients [24]. Female patients were reported to have a significantly higher prevalence of malnutrition than male patients [12]. Herein, the prevalence of malnutrition was also higher in female patients, but the difference was not significant.

Surgical intervention is indicated for UC when medical treatments are ineffective or intolerable because of side effects or when there is a life-threatening hemorrhage, toxic megacolon, or perforation [26]. Several previous studies have reported that the prevalence of malnutrition in UC patients undergoing surgery could reach 60–63% [27,28]. IBD-related surgery was associated with the risk of malnutrition [12,22], and patients with malnutrition had higher rates of surgical complications and higher rates of readmission and reoperation [29]. However, data on the association between baseline nutritional status and surgery during follow-up are sparse. Our study showed that the proportion of patients requiring surgical treatment at the first hospitalization or during the follow-up period was significantly higher in malnourished patients than in well-nourished ones, while the difference was not significant after adjusting for disease activity. Beyond the gastrointestinal tract, some IBD patients can also exhibit extra-intestinal manifestations (EIMs), such as mucocutaneous (erythema nodosum, pyoderma gangrenosum), ocular (episcleritis and uveitis), musculoskeletal (spondyloarthropathy and arthralgia), and hepatobiliary (primary sclerosing cholangitis and non-alcoholic fatty liver disease) manifestations [30]. Among the UC patients in this study, 16.7% suffered from at least one EIM, lower than a Greek study with a rate of 24.2% [31]. Although EIMs can reduce the quality of life [32], they were demonstrated to have no impact on the risk of malnutrition [22]. Consistently, we found no significant difference in the proportion of patients with EIMs between the malnutrition and non-malnutrition groups. However, considering the relatively small sample size of patients with EIMs in our study, the associations between EIMs and malnutrition need to be verified in the future with a larger sample size.

Moreover, CMV reactivation and *C. difficile* infection are important clinical issues in IBD, especially in UC patients, which can aggravate the symptoms of patients and are sometimes difficult to identify [33,34]. Several studies have analyzed the effects of CMV reactivation and *C. difficile* infection on IBD [35,36]. Nevertheless, to our knowledge, the present study is the first to explore the association between nutritional status and opportunistic infection in IBD patients. The immunosuppressives used in IBD can increase the risk of opportunistic infections. At the same time, deficiencies in protein and energy as well as micronutrients, such as zinc, vitamin D, and vitamin E, can also lead to infection susceptibility [37], and the worsened symptoms caused by infections might preclude patients from enough intake and result in a vicious cycle.

Most UC patients experience intermittent relapses in the first 3–7 years after diagnosis [38], while studies on the association between malnutrition and readmission in UC patients are limited. A previous study in IBD outpatients reported that the risk of an impaired nutritional status was correlated with the flare in the following three months, whereas the quantities of patients with an increased risk of impaired nutritional status (49 of 417 patients) and patients with disease flares (53 of 417 patients) were small, and separate analyses among IBD subtypes were not performed [39]. Herein, we analyzed the association between baseline nutritional status and readmission in UC patients who had regular follow-ups. We found that the period between the first and second hospitalization was significantly shorter in the initially malnourished patients, compared with well-nourished patients. Notably, multivariable analysis showed that malnutrition and disease activity were significantly associated with an increased risk of readmission, providing evidence for the potential predictive value of malnutrition diagnosed based on the GLIM for disease flares requiring hospitalization.

For UC patients with mild, moderate, or severe disease activity, HGB, ALB, and PA were significantly lower in patients with malnutrition. However, the difference in hsCRP between patients with and without malnutrition was only significant in the group with moderate disease activity, indicating that the inflammation level might be relatively even in patients with mild or severe disease activity, whether well-nourished or malnourished. Considering that the level of ALB can be affected by both nutrition and inflammation [40], the value of ALB as a nutritional indicator might be higher in UC patients with mild or severe disease activity, compared to those with moderate disease activity, and the inflammation level should be considered more when evaluating the nutritional status of patients with moderate disease activity.

The present study has several limitations. The retrospective nature of this study might have limited the power of our analysis, and the data from a single center might not be representative enough for the whole population of hospitalized UC patients. For example, the high proportion of patients with severe disease activity in our cohort might have led to a relatively high estimated prevalence of malnutrition. Therefore, further cross-sectional or prognosis studies involving multiple centers are required to verify the value of the GLIM criteria in diagnosing malnutrition in UC patients. Additionally, the energy intake could not be evaluated because of the lack of exact food intake and accurate intake of enteral nutrition formula in the electronic medical records. Thus, this study could not investigate the association between energy intake and malnutrition. The detailed collection of data on food intake or nutritional support is necessary for a future study, and apart from clinical manifestations, the nutritional status of patients should also be followed up after discharge to clarify the relationship between nutrition and prognosis more comprehensively.

## 5. Conclusions

The present findings suggested that a large part of hospitalized UC patients had malnutrition, according to the GLIM criteria, especially those with severe disease activity. Malnutrition was associated with opportunistic infections, tended to be associated with surgical treatment, and was a risk factor for readmission. Nutritional assessment deserves more attention in UC patients, and more studies are needed to confirm the prognostic value of malnutrition based on the GLIM criteria for UC in the future.

## Figures and Tables

**Table 1:** The comparison of demographics and clinical characteristics between patients with and without malnutrition according to the GLIM criteria.

	Total (*n* = 605)	Malnutrition (*n* = 388)	Non-Malnutrition (*n* = 217)	*p*-Value
Female (%)	263 (43.5%)	176 (45.4%)	87 (40.1%)	0.210
Age, years	41.3 ± 12.8	40.7 ± 13.2	42.5 ± 12.2	0.107
Age at diagnosis, years	35.7 ± 12.5	35.9 ± 13.3	35.2 ± 11.1	0.466
Body mass index, kg/m^2^	20.8 ± 3.5	19.5 ± 3.0	23.2 ± 2.9	<0.001
Disease duration, months	48 (12, 96)	36 (12, 84)	60 (24, 120)	<0.001
Duration of follow-up, days	459 (103, 1007)	455 (110, 1043)	486 (97, 970)	0.763
Length of stay, days	15 (9, 22)	18 (12, 26)	9 (6, 15)	<0.001
Disease Activity				<0.001
Mild (%)	114 (18.8%)	39 (10.1%)	75 (34.6%)	
Moderate (%)	265 (43.8%)	153 (39.4%)	112 (51.6%)	
Severe (%)	226 (37.4%)	196 (50.5%)	30 (13.8%)	
Disease extent				<0.001
E1(%)	25 (4.1%)	6 (1.5%)	19 (8.8%)	
E2(%)	99 (16.4%)	50 (12.9%)	49 (22.6%)	
E3(%)	481 (79.5%)	332 (85.6%)	149 (68.7%)	
Patients accompanied by opportunistic infection (%)	153 (25.3%)	126 (32.5%)	27 (12.4%)	<0.001
Patients with extra-intestinal manifestations (%)	101 (16.7%)	68 (17.5%)	33 (15.2%)	0.463
Patients treated with surgery (%)	79 (13.1%)	65(16.8%)	14 (6.5%)	<0.001

The data are presented as *n* (%), mean ± SD, or the median (Q1, Q3).

**Table 2:** The comparison of clinical characteristics and nutritional status between patients with and without readmission.

	Total (*n* = 516)	Patients with Readmission (*n* = 205)	Patients without Readmission (*n* = 311)	*p*-Value
Disease duration, months	48 (12, 96)	36 (12, 84)	48 (24, 120)	0.001
Disease Activity				<0.001
Mild (%)	93 (18.0%)	24 (11.7%)	69 (22.2%)	
Moderate (%)	229 (44.4%)	83 (40.5%)	146 (46.9%)	
Severe (%)	194 (37.6%)	98 (47.8%)	96 (30.9%)	
Disease extent				0.142
E1 (%)	18 (3.5%)	6 (2.9%)	12 (3.9%)	
E2 (%)	90 (17.4%)	28 (13.7%)	62 (19.9%)	
E3 (%)	408 (79.1%)	171 (83.4%)	237 (76.2%)	
Patients accompanied by opportunistic infection (%)	135 (26.2%)	61 (29.8%)	74 (23.8%)	0.132
Patients with extra-intestinal manifestations (%)	75 (14.5%)	37 (18.0%)	38 (12.2%)	0.066
Prevalence of malnutrition (%)	332 (64.3%)	156 (76.1%)	176 (56.6%)	<0.001

The data are presented as *n* (%) or the median (Q1, Q3).

**Table 3:** Multivariate logistic regression analysis of risk factors of readmission.

	OR	95% CI	*p*-Value
Malnutrition	1.738	1.128–2.677	0.012
Disease activity	1.457	1.102–1.927	0.008
Disease duration	0.996	0.993–0.999	0.011
Extra-intestinal manifestations	1.505	0.898–2.525	0.121

## Data Availability

The data underlying this study are available from the corresponding author upon reasonable request.
